# Triptycene Induced Enhancement of Membrane Gas Selectivity for Microporous Tröger's Base Polymers

**DOI:** 10.1002/adma.201305783

**Published:** 2014-03-14

**Authors:** Mariolino Carta, Matthew Croad, Richard Malpass-Evans, Johannes C Jansen, Paola Bernardo, Gabriele Clarizia, Karel Friess, Marek Lanč, Neil B McKeown

**Affiliations:** School of Chemistry, Cardiff UniversityCardiff, CF10 2AT, UK E-mail: neil.mckeown@ed.ac.uk; Institute on Membrane Technology, ITM-CNR, c/o University of CalabriaVia P. Bucci 17/C, 87036, Rende (CS), Italy E-mail: jc.jansen@itm.cnr.it; Institute of Chemical Technology, Department of Physical ChemistryTechnická 5, Prague 6, 166 28, Czech Republic

**Keywords:** polymer membranes, gas separation, triptycene, intrinsic microporosity

Polymer membranes are of increasing importance for a range of molecular separations due to their potential for enhanced energy-efficiency over competing technologies.^1^ For example, gas separation using polymer membranes is an established industrial technology that is applied to O_2_ or N_2_ enrichment of air, natural gas upgrading (i.e., predominantly removing CO_2_ from CH_4_), and hydrogen recovery from ammonia manufacture (separating H_2_ from N_2_).^2^ In addition, polymer membranes are also predicted to play an increasing role in hydrogen production (e.g., separating H_2_ from CO_2_)^3^ and post-combustion capture of CO_2_ (separating CO_2_ from N_2_).^4^ For any gas separation membrane it is desirable to have good selectivity for one gas over another, combined with high permeability (i.e., flux or throughput). Generally, polymers used for commercial gas separation membranes demonstrate high selectivity but low permeability, however, to compete with other technologies for very large-scale applications, membrane materials with greatly enhanced gas permeabilities are desirable.^5^ Unfortunately, for the separation of a given gas pair (*x* and *y*), highly permeable polymers display inadequate selectivity due to the well-established trade-off between permeability (*P_x_*) and selectivity (*α_xy_* = *P_x_/P_y_*). For example, one of the most permeable polymers, poly(trimethylsilylpropyne), has very poor selectivity (e.g., *PO_2_* = 6000 Barrer; *αO_2_/N_2_* = 1.8).^6^ The permeability-selectivity trade-off was quantified by Robeson in 1991,^7^ and revised in 2008,^8^ by plotting *log P_x_* versus *log α_xy_* for a large number of polymers. For each gas pair, an “upper bound” was identified and the position of the gas permeability data relative to the upper bound is used as a universal performance indicator when assessing the potential of a new polymer as a membrane material for the separation of the two gases. The position of the Robeson upper bound for each gas pair is related to the difference in the kinetic diameter of the gas molecules and the size-sieving nature (i.e., diffusivity selectivity) of the rigid glassy polymers that define it.^9^ Theory suggests that greater selectivity can be obtained by suppressing polymer motion by increasing chain rigidity whereas high gas permeability relies on the generation of a large amount of free volume through large inter-chain separation.[9b] Conforming to these design criteria, Polymers of Intrinsic Microporosity (PIMs), such as the archetypal PIM-1 (**Figure** **1**a), are a class of membrane-forming polymer that are designed to possess a wholly fused-ring structure to restrict chain motion and a contorted chain structure to prohibit space efficient packing.^10^ Hence, gas permeability data for PIMs generally lie over the 1991 upper bound for most important gas pairs and some approach, or even exceed, the 2008 upper bounds (**Figure** **2**).^11^

**Figure 1:**
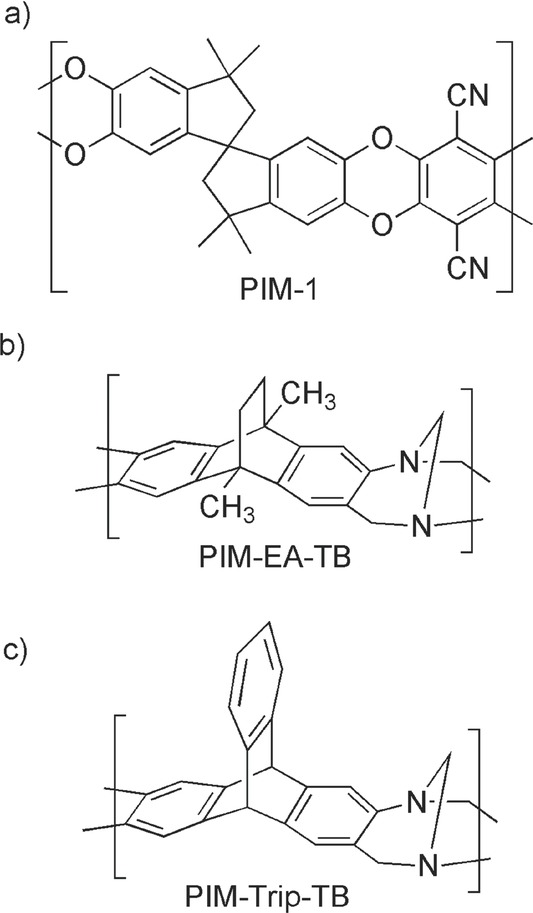
The molecular structures of a) PIM-1; b) PIM-EA-TB and c) PIM-Trip-TB.

**Figure 2:**
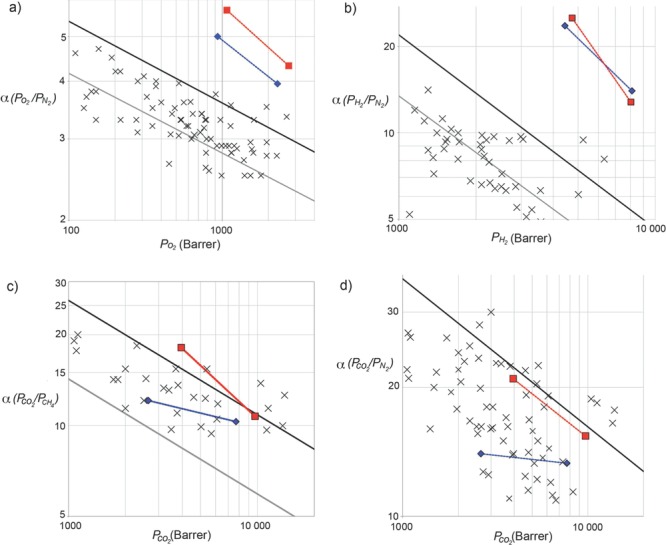
Robeson plots for a) O_2_/N_2_; b); H_2_/N_2_ c) CO_2_/CH_4_ and d) CO_2_/N_2_ gas pairs showing the data for methanol treated PIM-Trip-TB with data points 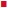 for a 132 μm film and 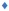 for PIM-EA-TB for a 180 μm film. Data at lower permeability, joined by a line, are for the same films but aged 100 and 470 days, respectively. The gray and black lines represent the 1991^10^ and 2008^11^ upper bounds, respectively. Also shown, as crosses, are data points of other PIMs reported since the upper bound was updated in 2008.

Recently, we introduced a new class of PIM prepared using a polymerisation reaction based on the efficient formation of the bridged bicyclic diamine called Tröger's base (TB; i.e., 6H,12H-5,11-methanodibenzo[b,f][1,5]diazocine).^12^ For example, the reaction of 2,6(7)-diamino-9,10-dimethylethanoanthracene yields the highly rigid ladder polymer (PIM-EA-TB) ( Figure 1b). PIM-EA-TB demonstrates very fast gas permeability and good selectivity so that its data lie well above the 2008 upper bounds for O_2_/N_2_ (Figure 2a), H_2_/N_2_ ( Figure 2b), H_2_/CH_4_ and H_2_/CO_2_ due to exceptional diffusivity selectivity that favours the transport of gas molecules of smaller kinetic diameters (e.g., H_2_ = 2.8; CO_2_ = 3.3; O_2_ = 3.46 Å) over that of larger molecules (e.g., N_2_ = 3.64; CH_4_ = 3.87 Å).[12a] This enhanced molecular sieving performance of PIM-EA-TB was attributed to its highly rigid macromolecular structure, which does not contain the relatively flexible spiro-centres or dioxin linkages found in conventional PIMs such as PIM-1.^13^ Anomalously, the permeability data of PIM-EA-TB for the technologically important gas pairs of CO_2_/CH_4_ and CO_2_/N_2_ proved unremarkable relative to that of other PIMs and fall below the 2008 upper bounds ( Figure 2b and c).[12a] Here we report the properties of a novel triptycene-based PIM prepared via Tröger's base formation that demonstrates further enhancement of gas permeability data for O_2_/N_2_ and performance near the 2008 upper bounds for the CO_2_/CH_4_ and CO_2_/N_2_ gas pairs.^14^

PIM-EA-TB was designed to possess extreme rigidity being composed solely of benzene rings fused to alternating TB and ethanoanthracene bridged bicyclic units. Therefore, it is a challenge to reduce polymer chain mobility still further. However, one possible enhancement to the polymer design is the removal of the methyl substituents at the bridgehead of the ethanoanthracene unit as their turnstile-like rotary thermal motion may reduce the gas selectivity of PIM-EA-TB by facilitating transport of larger gas molecules through narrow elements of free volume that would otherwise be inaccessible. These methyl groups were originally envisioned to increase the distance between polymer chains of PIM-EA-TB to ensure high permeability. Instead, triptycene was considered as a desirable alternative bicyclic building unit as it has been used extensively to generate free volume and intrinsic microporosity within polymers.^15^ The use of triptycene, to give PIM-Trip-TB ( Figure 1c), was anticipated to maintain inter-chain distances, without the need for bridgehead methyl substituents.

PIM-Trip-TB is prepared in three simple steps from commercially available triptycene. The final step involves polymerisation of 2,6(7)-diaminotriptycene^16^ by reaction with six equivalents of dimethoxymethane in trifluoroacetic acid (TFA) at ambient temperature. The resulting polymer is soluble in chloroform allowing for analysis by Gel Permeation Chromato­graphy (GPC) which indicates that a reasonably high average molecular mass was achieved (*M_n_* = 21 000; *M_w_* = 50 000; calibrated relative to polystyrene standards). An apparent BET (Brunauer, Emmett, Teller) surface area of 899 m^2^ g^−1^ could be calculated from the N_2_ isotherm for a powdered sample of PIM-Trip-TB at 77 K,^17^ which is less that that obtained for the related polymer PIM-EA-TB (1028 m^2^ g^−1^) but higher than the range quoted for PIM-1 (720–820 m^2^ g^−1^) in the literature.[10b],^18^ The fabrication of optically clear films suitable for gas permeability studies was achieved by casting from chloroform solution. Films of PIM-Trip-TB proved robust with a Young's modulus of 1206 ± 42 MPa and tensile strength of 44.6 ± 5.2 MPa, which are an improvement on the equivalent values for PIM-EA-TB,[12a] and maximum deformation of 13.0 ± 8.2%.

The gas permeabilities of a film of PIM-Trip-TB of 132 μm thickness are given in **Table** **1** together with the corresponding values for a thicker film of PIM-EA-TB (182 μm), which are similar to those recently reported.[12a] Prior to analysis both films were treated by immersion in methanol as this is known to reverse the effects of physical ageing for glassy ultra-permeable polymers and also removes the last residues of the casting solvent.[10d] Hence, this treatment allows a direct comparison between the gas permeabilities of the different polymers in an unaged state.

**Table 1:** The gas permeabilities P_x_, diffusivity D_x_, solubility coefficient S_x_ and ideal selectivities α (P_x_/PN_2_) for a methanol treated film of PIM-Trip-TB of thickness = 132 μm with comparable data for a the same film obtained after 100 days given in parentheses. To allow a direct comparison, data from a film of PIM-EA-TB (180 μm) (and aged for 470 day in parentheses), following an identical pre-treatment, is provided. P_x_ and D_x_ values were determined by time lag analysis, S_x_ was determined by gravimetric sorption measurements

	N_2_	O_2_	CO_2_	CH_4_	H_2_	He
*P_x_* (PIM-Trip-TB) [Barrer]	629 (189)	2718 (1073)	9709 (3951)	905 (218)	8039 (4740)	2500 (1585)
*α* (*P_x_/PN_2_*) (PIM-Trip-TB)	– (–)	4.3 (5.7)	15.9 (21.0)	1.4 (1.4)	12.8 (25.1)	4.0 (8.4)
*D_x_* (PIM-Trip-TB) [10^−12^ m^2^/s]	135 (28.5)	462 (148)	111 (34.6)	48.9 (7.5)	>7800 (4900)	>10000 a) (>7700)
*D_x_/DN_2_* (PIM-Trip-TB)	–	3.4 (5.2)	0.82 (1.3)	0.36 (0.26)	58 a) (172)	74 a) (270)
*S_x_* (PIM-Trip-TB) [cm^3^ cm^−3^ bar^−1^]	5.3	–	51.0	21.5	–	–
*S_x_/SN_2_* (PIM-Trip-TB)	–	–	9.6	4.1	–	–
*P_x_* (PIM-EA-TB) [Barrer]	580 (188)	2294 (933)	7696 (2644)	774 (219)	8114 (4442)	2685 (1630)
*α* (*P_x_/PN_2_*) (PIM-EA-TB)	– (–)	3.95 (4.95)	13.3 (14.1)	1.3 (1.2)	14.0 (23.6)	4.6 (8.7)
*D_x_* (PIM-EA-TB) [10^−12^ m^2^/s]	89 (22.9)	310 (104)	76.4 (35.2)	31.9 (6.9)	>7200 (4000)	>10000 a) (>7700)
*D_x_/DN_2_* (PIM-EA-TB)	–	3.48 (4.5)	0.82 (1.54)	0.36 (0.30)	81 a) (174)	113 a) (337)
*S_x_* (PIM-EA-TB) [cm^3^ cm^−3^ bar^−1^]	4.4	–	47.0	17.2		
*S_x_/SN_2_* (PIM-EA(Me)-TB)	–	–	10.8	3.9	–	–

a) For He and H_2_ the time lag is too short (<1 s) for absolute determination of *D* and the indicated value of *D* is the minimum limit.

The order of gas permeabilities for the freshly methanol treated film of PIM-Trip-TB is CO_2_>H_2_>O_2_>He>CH_4_>N_2_, which is different from that of PIM-EA-TB (H_2_>CO_2_>He>O_2_>CH_4_>N_2_) but similar to most other PIMs. Greater values of gas permeabilities are obtained for PIM-Trip-TB over PIM-EA-TB with the exception of He and H_2_, which are similar. This enhancement is particularly notable as the film of PIM-Trip-TB was thinner than that of PIM-EA-TB and, generally for glassy polymers, the gas permeabilities for thinner films are lower due to the larger relative contribution of the more densely packed surface regions of the film.[11a],^19^ Of particular interest is the greater CO_2_ permeability for PIM-Trip-TB. In addition, ideal selectivities for PIM-Trip-TB are significantly higher than those of PIM-EA-TB for some important gas pairs (O_2_/N_2_, CO_2_/CH_4_ and CO_2_/N_2_) whilst similar for others (H_2_/N_2_, H_2_/CO_2_ and H_2_/CH_4_). Hence, the permeability data for PIM-Trip-TB are well above the Robeson upper bounds for O_2_/N_2_ ( Figure 2a), H_2_/N_2_ ( Figure 2b), H_2_/CH_4_ and H_2_/CO_2_. In particular, the strong enhancement in O_2_/N_2_ performance vindicates the choice of triptycene as a structural unit to enhance diffusivity selectivity as the separation of this gas pair relies almost exclusively on size selectivity based on only a small difference in kinetic dia­meter between the gas molecules. Importantly, the PIM-Trip-TB data for CO_2_/CH_4_ ( Figure 2c) and CO_2_/N_2_ ( Figure 2d), both lie very close to the respective 2008 upper bounds in contrast to that of PIM-EA-TB, which fall well below.

Physical ageing (i.e., loss of free volume over time) is a general feature of glassy polymers^20^ and is observed for both PIM-Trip-TB and PIM-EA-TB (Table 1). The rate of ageing is more rapid in thinner films and therefore, to allow direct comparison, the values quoted are for a 60% reduction of the original value of PO_2_, which occurs after 100 days following methanol treatment for the thinner film of PIM-Trip-TB (132 μm) and 470 days for that of PIM-EA-TB (180 μm). PIM-Trip-TB ages gracefully with a corresponding enhancement of selectivity for the reduction in permeability so that lines joining the non-aged and aged data points on all the Robeson plots are approximately parallel to the upper bound for all important gas pairs ( Figure 2). For the O_2_/N_2_ gas pair, the selectivity in favour of O_2_ of 5.7 for a value of PO_2_ greater than 1000 Barrer is exceptional. In addition, the data for the aged film of PIM-EA-TB crosses the 2008 upper bound for CO_2_/CH_4_ (Figure 2c) and is comparable with the data of the best performing PIMs for this commercially important gas pair.[11a]

According to the solution-diffusion model the permeability of a gas through a dense polymer is dependent on both its diffusivity (*D_x_*) and its solubility (*S_x_*) (i.e., *P_x_* = *D_x_S_x_*).^21^ Generally, the observed enhancement in performance for PIM-Trip-TB as compared to PIM-EA-TB appears to be due to greater diffusivity selectivity as determined by analysis of the data obtained by the time-lag method for measuring gas permeabilities. This is consistent with the higher chain stiffness as expressed in a higher Young's modulus. In order to investigate the mechanism of transport in these two polymers further, CO_2_, CH_4_ and N_2_ sorption isotherms were obtained at 25 °C (**Figure** **3**a). The uptake of ∼20% (∼4 mmol g^−1^) by mass of CO_2_ at 0.7 MPa for these TB-based polymers is impressive as compared to other micro­porous polymers examined for CO_2_ adsorption.^17^,^22^ Interestingly, the sorption of CO_2_, CH_4_ and N_2_ within PIM-Trip-TB is higher at relatively low pressures (0.1–0.3 MPa) as compared to PIM-EA-TB, thus contributing to their higher solubility coefficients and, hence, gas permeabilities at the feed pressure of 0.1 MPa (Table 1). In contrast, the sorption of CO_2_ and CH_4_ is greater at higher pressures (>0.3 MPa) for PIM-EA-TB relative to PIM-Trip-TB ( Figure 3b). This difference in sorption behaviour indicates that PIM-Trip-TB has a higher proportion of smaller free volume elements that are capable of binding to gas molecules at lower pressures than does PIM-EA-TB, which is consistent with its enhanced size-sieving properties.

**Figure 3:**
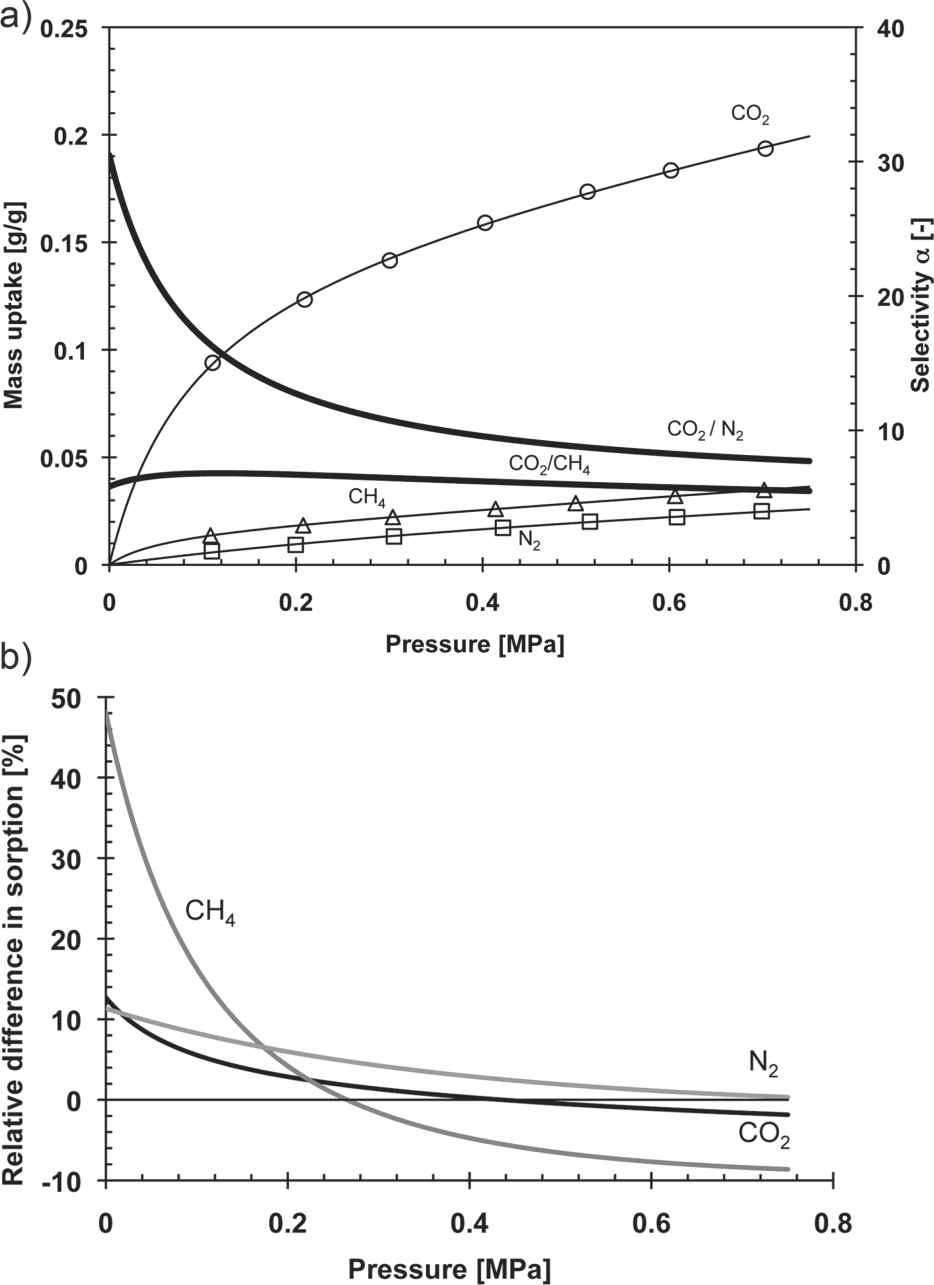
a) Sorption isotherms for CO_2_, CH_4_ and N_2_ in MeOH treated PIM-Trip-TB film of 132 μm thickness. Thin solid lines represent the fit of the data according to the dual mode sorption model and thick solid lines represent the corresponding ideal sorption selectivity calculated from the curve fit. b) Relative differences in sorption of CO_2_, CH_4_ and N_2_ between MeOH treated films of PIM-Trip-TB and PIM-EA-TB. Positive values indicate a pressure where gas sorption in PIM-Trip-TB is higher than that of PIM-EA-TB.

Despite the clear enhancement noted for Trip-PIM-TB, the performance of both TB polymers appears anomalous for gas pairs involving CO_2_ with the expected benefits of enhanced diffusivity selectivity, which are obvious for other gas pairs, not being fully realised for CO_2_/CH_4_ or CO_2_/N_2_. In particular, the apparent diffusivity selectivity in favour of N_2_ over CO_2_ within unaged films of both Trip-PIM-TB and PIM-EA-TB (Table 1) is unusual and suggests that some of the free volume of PIM-EA-TB is blocked for CO_2_ transport but available for N_2_ and other gases. It is known that adsorbed water adversely effects the gas permeability of PIM-1[10d] and therefore, to determine whether its presence may be influencing the performance of the TB polymers, IR spectroscopic analysis was carried out on films of PIM-EA-TB and PIM-Trip-TB following methanol treatment. Characteristic absorption bands of H_2_O at 3200–3600 cm^−1^ and 1630 cm^−1^ were observed in the spectrum of both films (Figure SI 1). In addition, a relatively strong and sharp band at 3650 cm^−1^ can be assigned to the presence of hydroxide anion, consistent with the basic nature of the TB units within the polymers.^23^,^24^ Prior to the gas permeability measurements, the polymer films were subjected to reduced pressure (10^−2^ mbar) and hence, the IR spectra of films following a similar protocol were also collected. A distinct difference between the spectra of the resulting two films was observed with that of PIM-Trip-TB showing a large reduction (∼95%) in the intensity of the broad water peak centred at 3400 cm^−1^ whereas the intensity of the same peak for PIM-EA-TB is reduced by only ∼35%. Similarly, after exposure to reduced pressure, the intensity of the peak attributed to the hydroxide anion at ∼3650 cm^−1^ is reduced to 20% of its original intensity for PIM-Trip-TB but it is unchanged for PIM-EA-TB. Hence, the anomalously low permeability of CO_2_ relative to that of other gases may be due to its specific interaction with adsorbed water and associated hydroxide anions to form bicarbonate anions that will occupy free volume and reduce diffusivity. This behaviour is similar to that noted for another recently reported amine-containing PIM.^25^ However, PIM-Trip-TB appears to offer an additional advantage over PIM-EA-TB in that it is less hydroscopic and therefore water does not have such a significant effect on its CO_2_ permeability.

To conclude, PIM-Trip-TB provides highly impressive data for a number of commercially relevant gas pairs including O_2_/N_2_, H_2_/N_2_ and CO_2_/CH_4_ in terms of permeability, selectivity and position relative to the Robeson upper bound. This performance is based on exceptional diffusivity selectivity of gas transport. The lack of methyl groups in PIM-Trip-TB may enhance performance as compared to PIM-EA-TB, within which methyl rotary motion may reduce diffusivity selectivity. Indeed, as PIM-Trip-TB is a ladder polymer that is composed of only benzene rings fused together by rigid bridged bicyclic units and contains no groups that can act as rotors, its performance as a molecular sieve is likely to be approaching the optimum achievable for gas separation for a solution processable polymer. Any further improvement in fundamental gas permeability data may require the additional enhancement of solubility selectivity for one gas over another.

## Experimental Section

*Preparation of PIM-Trip-TB*: A mixture of 2,6(7)-diaminotriptycene (1.43 g, 5.0 mmol), dimethoxymethane (2.60 g, 34.2 mmol) and dichloromethane (1.5 mL) was added dropwise to trifluoroacetic acid (15 mL) over 2 hours. The clear red solution was left stirring for 168 hours and the reaction quenched with water (100 mL). Aqueous ammonia (35%, 100 mL) was added and the mixture stirred vigorously for 2 hours before the precipitate was collected by filtration. The solid was washed with water (100 mL) and acetone (100 mL), dried and ground to a fine powder. The polymer was dissolved in chloroform (150 mL), reprecipitated with hexane (150 mL) and collected by filtration. This process was repeated twice. The solid was then refluxed in acetone and twice in methanol, each time for 16 hours. Filtration gave the polymer as a cream powder (1.28 g, 79%). Apparent BET surface area of powder = 899 m^2^ g^−1^; total pore volume = 0.55 ml g^−1^ at *p/p^o^* = 0.98; TGA (nitrogen): weight loss due to thermal degradation started at 400 °C and totalled 30.5%; GPC (from chloroform solution and calibrated against polystyrene standards) *M_n_* = 21,200, *M_w_* = 50,700 g mol^−1^; ^1^H NMR (400 MHz, CDCl_3_) *δ* ppm 3.89 (br s, 2H), 4.41 (br s, 2H), 4.80 (br s, 2H), 5.07 (br s, 2H), 6.97 (br m, 8H).

*Procedures*: Film formation was achieved by preparing a solution of PIM-Trip-TB (0.50 g) in chloroform (20 mL) which was poured into a 9 cm circular Teflon mould. The film was allowed to form by slow solvent evaporation for 96 h. Prior to permeability measurements the films were soaked in methanol for 8 h to remove residual casting solvent and then dried in air. The density of the 132 μm film, measured by simple geometric means, was found to be 1.14 ± 0.04 g cm^−3^ after MeOH soaking. Gas permeation tests of single gases were carried out at 25 °C and at a feed pressure of 1 bar, using a fixed-volume pressure increase instrument, described elsewhere,^26^ with details given in the Supplemental Information. Before analysis the membrane samples were carefully evacuated to remove previously dissolved species using a vacuum pump fitted with a trap to remove oil. The gases were tested in the following order: He, H_2_, N_2_, O_2_, CH_4_, CO_2_. An effective membrane area of 2.14 cm^2^ was used.

## Supporting Information

As a service to our authors and readers, this journal provides supporting information supplied by the authors. Such materials are peer reviewed and may be re-organized for online delivery, but are not copy-edited or typeset. Technical support issues arising from supporting information (other than missing files) should be addressed to the authors.
