# Supplementary material for: Triptycene Induced Enhancement of Membrane Gas Selectivity for Microporous Tröger's Base Polymers
Source: Adv Mater. 2014 Mar 14;26(21):3526–31. doi: 10.1002/adma.201305783 (PMC4223990; doi:10.1002/adma.201305783)
Supplement: Supplementary file 1 — Supplementary [file adma0026-3526-SD1.pdf]

Copyright WILEY-VCH Verlag GmbH & Co. KGaA, 69469 Weinheim, Germany, 2014.

ADVANCED MATERIALS

Supporting Information

for *Adv. Mater.*, DOI: 10.1002/adma.201305783

Triptycene Induced Enhancement of Membrane Gas Selectivity for Microporous Tröger's Base Polymers

Mariolino Carta, Matthew Croad, Richard Malpass-Evans, Johannes C. Jansen, Paola Bernardo, Gabriele Clarizia, Karel Friess, Marek Lanč, and Neil B. McKeown**

Submitted to ADVANCED MATERIALS

DOI: 10.1002/adma.201305783

Triptycene induced enhancement of membrane gas selectivity for microporous Tröger's base polymers

By Mariolino Carta, Matthew Croad, Richard Malpass-Evans, Johannes C. Jansen, Paola Bernardo, Gabriele Clarizia, Karel Friess, Marek Lanč and Neil B. McKeown**

Supplemental Information

1. Materials

1.1. General methods and equipment

Commercially available reagents were used without further purification. Anhydrous dichloromethane was obtained by distillation over calcium hydride under nitrogen atmosphere. Anhydrous *N*,*N*-dimethylformamide was bought from Aldrich. All reactions using air/moisture sensitive reagents were performed in oven-dried or flame-dried apparatus, under a nitrogen atmosphere. TLC analysis refers to analytical thin layer chromatography, using aluminium-backed plates coated with Merck Kieselgel 60 GF_{254}. Product spots were viewed either by the quenching of UV fluorescence, or by staining with a solution of Cerium Sulfate in aqueous H_2SO_4. Flash chromatography was performed on silica gel 60A (35-70 micron) chromatography grade (Fisher Scientific). Melting points were recorded using a Gallenkamp Melting Point Apparatus and are uncorrected. Infra-red spectra were recorded in the range 4000-600 cm^{-1} using a Perkin-Elmer 1600 series FTIR instrument either as a thin film or as a nujol mull between sodium chloride plates. All absorptions are quoted in cm^{-1}. ^{1}H NMR spectra were recorded in the solvent stated using an Avance Bruker DPX 400 (400 MHz) or DPX 500 (500 MHz) instruments, with ^{13}C NMR spectra recorded at 100 MHz or 125 MHz respectively. Low-resolution mass spectrometric data were determined using a Fisons VG Platform II quadrupole instrument using electron impact ionization (EI) unless otherwise stated. High-resolution mass spectrometric data were obtained in electron impact ionization (EI) mode unless otherwise reported, on a Waters Q-TOF micromass spectrometer. Low-temperature (77 K) N_2 adsorption/desorption measurements of PIM

powders were made using a Coulter SA3100. Samples were degassed for 800 min at 120 °C under high vacuum prior to analysis. The TGA was performed using the device Thermal Analysis SDT Q600 at a heating rate of 10 °C/min from room temperature to 1000 °C. Elemental analysis was carried out with a Carlo Erba EA1101 elemental analyser. Tensile tests were carried out at room temperature on a Zwick/Roell single column Universal Testing Machine, model Z2.5, equipped with flat grinded steel pneumatic clamps, a 50N load cell and a 1 μm step resolution. Specimens with an effective length of 30 or 40 mm and a width of 5.0 mm were tested at a deformation rate of 3 or 4 mm min^{-1}, corresponding to 10 % min^{-1}. The Young's modulus, the tensile strength and the maximum deformation were determined as the average and the standard deviation of four samples. The thickness was determined as the average over the entire length, measured at 5 mm intervals, using a digital micrometer (Mitutoyo, model IP65). The sample weight was measured with a 5 decimals analytical balance and the geometrical density was determined directly from the ratio mass/volume. Mechanical tests were performed on the samples treated in MeOH and dried at RT.

2. Measurement of gas transport properties.

2.1 Pure gas permeation

Gas permeation rate measurements of single gases were carried out at 25 °C and at a feed pressure of 1 bar, using a fixed-volume pressure increase instrument, described elsewhere.[1]

Before analysis the membrane samples were carefully evacuated to remove previously dissolved species. The gases were tested in the following order: He, H_2, N_2, O_2, CH_4, CO_2. An effective membrane area of 2.14 cm^2 was used. The pressure increase in the permeate volume was monitored by a pressure transducer, starting from the instant of exposure of the membrane to the feed gas. Permeability coefficient, *P*, was calculated from the slope of the pressure-time curve in the steady state condition:

$$p_t = p_0 + \left(dp/dt\right)_0 \cdot t + \frac{RT \cdot A}{V_P \cdot V_m} \cdot \frac{p_f \cdot P}{l} \left(t - \frac{l^2}{6D} \right) \quad (1)$$

in which p_t is the permeate pressure at time t, p_0 the starting pressure, $(dp/dt)_0$ the baseline slope, p_f the feed pressure, R the universal gas constant, T the absolute temperature, A the exposed membrane area, V_P the permeate volume, V_m the molar volume of the permeating gas at standard temperature and pressure (0 °C and 1 atm). The term p_0 + $(dp/dt)_0$ accounts for the starting pressure and the baseline slope and is normally negligible in the case of well evacuated defect free samples.

The diffusion coefficient, D, of each gas through the membrane samples was calculated according to the time lag method:

$$D = \frac{l^2}{6\theta} \quad (2)$$

where l is the membrane thickness and θ is the gas time lag. Assuming the validity of the solution-diffusion transport mechanism, the solubility coefficient, S, was obtained indirectly via the equation:

$$S = P / D \quad (3)$$

Separation of gas pairs in polymeric membranes is defined by the permselectivity, obtained as the ratio of the permeability for two gases:

$$\alpha_{x/y} = P_x/P_y \quad (4)$$

Selectivity can be decoupled into solubility-selectivity and diffusivity-selectivity

$$\alpha_{x/y} = S_x/S_y \times D_x/D_y \quad (5)$$

The thickness of each membrane sample was obtained as average of at least six measurements by means of a digital micrometer (Mitutoyo).

2.2 Sorption analysis

Gas sorption experiments were performed gravimetrically using a self-developed sorption apparatus located in a temperature-controlled chamber at 25.0 ± 0.1°C. Apparatus was equipped with a calibrated McBain quartz spiral balance and with an automatic charge-coupled device (CCD, Sony) camera system detection of sample-target-point position. The detailed experimental procedure and a buoyancy testing were described previously.[2,3] The sample (flat membrane or powder in aluminium basket) was appended on the quartz spring. The glass measuring chamber was evacuated before each measurement to a pressure lower than 10^{-3} mbar by a rotary oil pump (Trivac D4B, Oerlikon Leybold). A Leybold oil-mist filter eliminated (with 99.99% efficiency) contamination of the measuring chamber with oil vapours from the pump. The sorption experiments were performed at the absolute pressure ranging from 0 to 8 bar. After the exposure of the sample to a particular gas at a known pressure, the elongation of the quartz spiral was monitored by an automatic optical system until the equilibrium state was reached. The average error of the mass determination reached approximately 30 micrograms.

The experimental data were fitted by the dual mode sorption model which describes sorption of gaseous penetrants in glassy polymers [4]:

$$c = k_D p + \frac{C_H b p}{1 + bp} \qquad (6)$$

where c is the sorbate concentration, p the sorbate pressure, k_D is the Henry's law constant, C_H is the Langmuir (monolayer) sorption capacity constant and b is the Langmuir affinity constant.

The slope of the isotherms at low pressure defines the infinite dilution solubility, wich can be calculted from the dual mode parameters:

$$S_0 = \lim_{p \to 0} \frac{dc}{dp} = k_D + C_H\, b \qquad (7)$$

3. IR Spectroscopic analysis

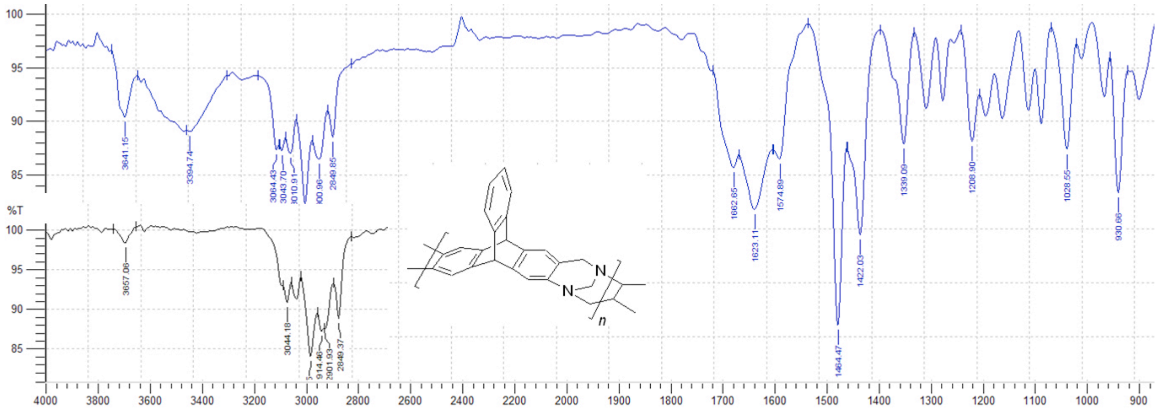

Fig. SI 1. IR spectra of a film of PIM-Trip-TB as cast and after exposure to low pressure (10^{-2} bar) for 24 h (inset below). Note the strong reduction of the intensity of peaks at 3395 cm^{-1} (water) and 3640 cm^{-1} (OH^{-}).

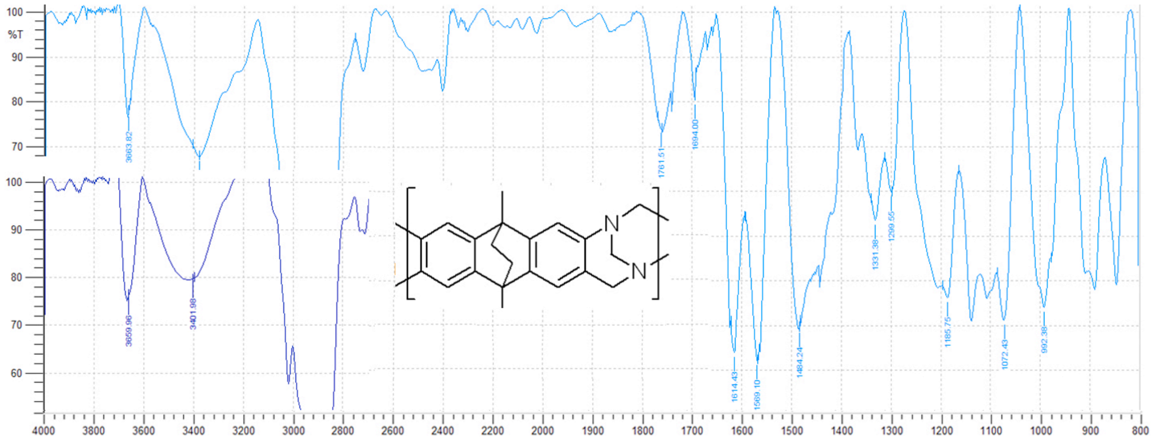

Fig. SI 2. IR spectra of a film of PIM-EA-TB as cast and after exposure to low pressure (10^{-2} bar) for 24 h (inset below). Note the retained intensity of peaks at 3395 cm^{-1} (water) and 3640 cm^{-1} (OH^{-}) after treatment.

References

[1] J. C. Jansen, K. Friess, E. Drioli, *J. Membr. Sci.* **2011**, *367*, 141.

[2] Vopička, K. Friess, V. Hynek, P. Sysel, M. Zgažar, M. Šípek, K. Pilnáček, M. Lanč, J.C. Jansen, C.R. Mason and P.M. Budd, *J. Membr. Sci.*, 2013, **434**, 148.

[3] K. Friess, V. Hynek, M. Šípek, W. M. Kujawski, O. Vopička, M. Zgažar and M. W. Kujawski, *Sep. Pur. Tech.*, 2011, **80**, 418.

[4] W.R. Vieth and K.J. Sladek, *J. Colloid Sci.* 1965, **20**, 1014.
